# Role of crocin in several cancer cell lines: An updated review

**DOI:** 10.22038/IJBMS.2019.37821.8995

**Published:** 2020-01

**Authors:** Ali Veisi, Ghaidafeh Akbari, Seyyed Ali Mard, Gholamreza Badfar, Vahid Zarezade, Mohammad Ali Mirshekar

**Affiliations:** 1 Behbahan Faculty of Medical Sciences, Behbahan, Iran; 2 Medicinal Plants Research Center, Yasuj University of Medical Sciences, Yasuj, Iran; 3 Research Center for Infectious Diseases of Digestive System [Alimentary Tract Research Center], Physiology Research Center (PRC), Department of Physiology, School of Medicine, Ahvaz Jundishapur University of Medical Sciences, Ahvaz, Iran; 4 Department of pediatrics, Behbahan Faculty of Medical Sciences, Behbahan, Iran; 5 Department of Physiology, School of Medicine, Zahedan University of Medical Sciences, Zahedan, Iran

**Keywords:** Cancer, Cell line, Crocin, Review, Tumor

## Abstract

Cancer is a major public health problem worldwide. The most important considerable features of cancer cells are uncontrolled proliferation, up-regulated differentiation, and immortality. Crocin, as a bioactive compound of saffron and as a water-soluble carotenoid has radical scavenging, anti-hyperlipidemia, memory improving, and inhibition of tumor growth effects. The present review was designed to evaluate molecular mechanisms underlying crocin effects against cancer cell lines. Data of this review have been collected from the scientific articles published in databases such as Science Direct, Scopus, PubMed, and Scientific Information Database from 1982 to 2019. According to various literature, crocin inhibits tumor growth, and its spread in several types of cancer including colorectal, pancreatic, breast, and prostate, as well as chronic myelogenous and leukemia. It inhibits telomerase activity, microtubule polymerization, cyclin D1, nuclear factor kappa B (NF-kB), multidrug resistance-associated protein (MRP1), and MRP2 overexpression. Crocin can induce apoptosis through activation of caspase 8, up-regulation of p53 expression, Bax/Bcl-2 ratio, and down-regulation expression of Bcl-2, survivin, and cyclin D1. It also down-regulates matrix metalloproteinase 2 and 9 (MMP2 and MMP9), N-cadherin, and beta-catenin expression, which are involved in tumor invasion and metastasis. Tumor invasion was also inhibited by crocin through increasing E-cadherin expression, cell cycle suppression at G1, G0/G1, S, and G2/M phases. Crocin has therapeutic and preventive effects on cancer cells line. Therefore, it has been suggested that this agent can be administered in patients that suffer from this problem.

## Introduction

Cancer is a major public health problem worldwide. Currently, cancer is the second leading cause of death and its incidence is expected to be more than heart disease in upcoming years (1). The most important considerable features of cancer cells are uncontrolled proliferation, up-regulated differentiation, and immortality (2). A great deal of *in vitro* and *in vivo* studies has reported that crocin has anti-cancer effects (Table 1). Here, we further interpreted and explained the role of crocin in genomic and molecular parameters in multiple cancer cell lines. 

**Figure 1:**
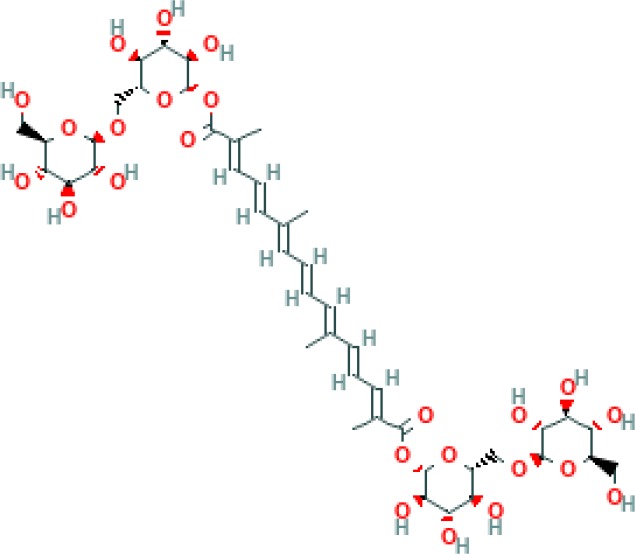
Chemical structure of crocin (C_44_H_64_O_24_)

Crocin, is a color agent (3) and water-soluble carotenoid pigment of the stigmas of *Crocus sativus *L. (4-6). There are four chemical analoges of crocin, including crocins 1–4. All of these analoges are glycosides of trans-crocetin, as a carotenoid derivative. Among the four above mentioned crocins, crocin 1 (crocin; alpha-crocin; crocetin digentiobiose ester) is the most abundant in saffron and has been extensively studied for its pharmacological effects (7). Crocin with the chemical structure of C_44_H_64_O_24_ (Figure 1) is the major reddish yellow pigment of saffron (8). 

Pharmacokinetic properties of crocin indicated that this agent is not completely absorbed into the blood by oral administration and excreted largely through the intestinal tract (9) due to hydrolysis to crocetin before or during intestinal absorption (10). 

***Biological activities of crocin***

Crocin physically binds to a wide range of cellular proteins such as structural proteins, membrane transporters, and enzymes involved in adenosine triphosphate (ATP) synthesis, redox homeostasis, and signal transduction (11). It has been shown that crocin increases glutathione synthesis and endogenous defense against oxidative stress (12). Other study indicated that oral administration of crocin-1 enhanced superoxide dismutase (SOD), and total antioxidant capacity in kidney tissue (13). Experimental studies have demonstrated that crocin promotes metal chelation (14) and scavenges free radicals (15). Recently, it has been shown that crocin has protective effects on cardiac injury following liver ischemia/reperfusion (I/R) damage through increasing anti-oxidants and modulating hemodynamic parameters (16). Furthermore, it has been reported that crocin has protective property in nephrotoxicity-induced by gentamicin (17), renal, hepatic, skeletal muscle, cardiac, gastric and brain I/R insult (18).

Anti-inflammatory and analgesic effects are other beneficial impacts of crocin (19). The anti-inflammatory properties of crocin are evident in studies that have shown a dual inhibitory effect *in vitro* against both cyclooxygenase 1 and 2 enzymes and prostaglandin E2 production. Pretreatment with crocin dose-dependently inhibited the xylene-induced ear edema in mice and carrageenan-induced paw edema in rats (20). Furthermore, crocin pretreatment reduced leukocyte infiltration, intercellular adhesion molecule 1(ICAM-1), and tumor necrotic factor-alpha (TNF-α) mRNA expression levels in I/R induced renal injuries in rats (21). In addition, it inhibited inducible nitric oxide synthase (iNOS) expression and nitric oxide production via down-regulation of nuclear factor kappa B (NF-kB) activity in lipopolysaccharide (LPS)- stimulated RAW 264.7 macrophages (22). Another study showed that crocin ameliorates proinflammatory cytokine levels including interleukin-1β (IL-1β), TNF-α, and IL-6 following venomous snakebite (23).

 It suppresses type 2 T helper cell chemokines via blocking extracellular signal-regulated kinases (ERK)- mitogen-activated protein kinases (MAPK)/NF-kB/ signal transducer and activator of transcription1 (STAT1) signaling pathways inTNF-a/ interferon (IFN)-c-stimulated human epidermal keratinocytes (24). The authors also suggest that crocin can suppress LPS-stimulated expression of iNOS by inducing hemoxygenase-1 (HO-1) expression via Ca^2+^calmodulin-Ca2^+^/calmodulin-dependent protein kinase 4 (CAMK4)- phosphoinositide 3-kinase (PI3K)/Akt (protein kinase B)-nuclear factor erythroid 2–related factor 2 (Nrf2) signaling cascades (22).

Besides, crocin can induce Ca^2+^ mobilization from intracellular pools and phosphorylation of CAMK4 in macrophages (25). This agent has anti-depressant-like action by increasing cAMP response element binding (CREB) protein, a transcription factor, BDNF (brain-derived neurotrophic factor), and VGF (a neuropeptide that may play a role in regulating energy homeostasis, metabolism, and synaptic plasticity) levels in the hippocampus (26). Crocin also has positive effects in preventing the impairment of learning and oxidative stress damage induced by chronic stress (27). Furthermore, crocin decreased D-galactose-induced memory and synaptic dysfunction, via attenuating ROS and advanced glycation end products (AGEs) formation (28). This natural product also has protective effects on tardive dyskinesia following administration of halluperidole (29).

Administration of crocin at higher doses over a 16-week period can prevent ovariectomy-induced osteoporosis without hyperplastic effects on the uterus (30). These reports imply that crocin also may have a potential inhibitory hormone-related cancers effect.

## Materials and Methods

Data of this review have been collected from the scientific articles published in databases such as Science Direct, Scopus, PubMed, and Scientific Information Database from 1982 to 2019.

## Results

***Molecular mechanisms of crocin on several cancer cell lines***

**Figure 2:**
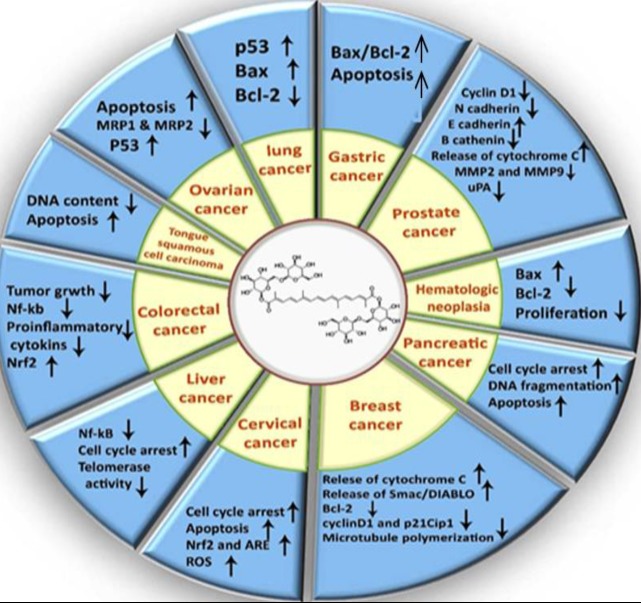
Some molecular mechanisms of crocin on several cancer cell lines

According to literature (Table 1), crocin through multiple mechanisms had regulatory effects on several cancer cell lines, which is in summary indicated in Figure 2. 

***Colorectal cancer***

Colorectal cancer (CRC) is a common gastrointestinal tract malignancy in the digestive tract following gastric and esophageal cancer. Rectum or the boundary between rectum and sigmoid colon are the most common sites for the occurrence of this type of cancer (31, 32). It has been shown that crocin reduces proliferation rate in human colorectal cancer cell lines HCT-116, HT-29, and SW-480, with the most significant anti-proliferative effect on HCT-116 cells (33). Crocin also exhibits anti-proliferative effect on both HCT116 wild-type and HCT116 p53−/− cell colon carcinoma cell lines (34). Documents show that chronic inflammation is one of the most important underlying conditions for tumor development (35). A classic example is CRC, which demonstrates a close correlation between chronic inﬂammation and carcinogenesis (36).

In the same regard, nuclear factor kappa-light-chain-enhancer of activated (NF-kB) transcription factor, a hallmark of inflammatory responses (37), which is frequently detected in tumors (38). Some evidence has suggested that NF-kB plays a major role in the progression of various human cancers. To some extent, non-steroidal anti-inflammatory drugs (NSAIDs) or glucocorticoids exert their anti-cancer effects through inhibition of NF-kB (39). 

Nrf2 is another factor that contributes to inflammation. In fact, Nrf2 is a transcription factor that regulates the expression of anti-oxidant elements and therefore protects organs against oxidative damage caused by inflammation and injury (40).

It has been suggested that animals treated with dextran sulfate sodium (DSS) undergo colitis, whereas those treated with both azoxymethane (AOM) and DSS undergo CRC. It has been shown that disruption of the Nrf2 gene increases susceptibility to DSS-induced colitis and to AOM-DSS-induced colon carcinogenesis (41, 42). Disruption of the Nrf2 gene increases the incidence of colonic tumor. Therefore, the Nrf2 pathway may mediate a significant anti-inﬂammatory response (43, 44). Some anti-inflammatory effects of crocin occur through inhibition expression and activation of NF-κB (19, 45). It has been demonstrated that crocin attenuates inflammation-associated liver cancer by inhibition of the NF-κB factor (46).

Crocin also inhibits DSS-induced colitis and decreases the mRNA expression of certain proinﬂammatory cytokines and increases Nrf2 expression in the colorectal mucosa. These findings suggest that crocin can suppress colitis, possibly by inhibiting inflammation (47).

***Breast cancer***

Breast cancer is the most common type of cancer and the first leading cause of cancer mortality in women worldwide. Genes involved in breast cancer can broadly be categorized into three groups: high, moderate, and low penetrance of clinical manifestation. In individuals carrying high-penetrance allele lifetime risks of breast cancer is above 50%. This percentage is greater than 20% in moderate penetrance alleles and about 10–20% in low-penetrance alleles (58). It has been reported to exhibit DNA fragmentation, down-regulate anti-apoptotic Bcl-2, and simultaneously up-regulate Bax, a proapoptotic Bcl-2 family molecule, in breast cancer cells (MCF-7). Crocin increases the release of cytochrome c, expression and activation of caspase 8, 9, and induces the cleavage of caspase-3 in MCF-7 cells (59). Furthermore, crocin significantly induces apoptosis through activation of caspase-8, up-regulation of Bax, and disruption of mitochondrial membrane potential in this cancer cell line (60).

Apoptosis induced by caspase occurs in a large degree by two pathways, caspase-9-dependent mitochondria-mediated apoptosis (endogenous) and caspase-8-dependent death receptor apoptosis (exogenous) (61). Activation of mitochondrial pathway leads to opening of mitochondrial permeability transition pore, decreased mitochondrial membrane potential and release of sequestered proapoptotic proteins such as cytochrome c, and second mitochondria-derived activator of caspase/direct inhibitor of apoptosis protein (IAP)-binding protein with low isoelectric point (Smac/DIABLO) from intermembrane space into the cytosol. Cytochrome c stimulates apoptosome formation followed by activation of caspase-9 and then the activation of the effector, caspase-3 (62, 63).

In cytosol, Smac/DIABLO interacts and antagonizes IAPs, thus allowing the activation of caspases and apoptosis. Caspase-8 activates crosstalk between the two pathways by cleavage of Bid, a BH3-only pro-apoptotic member of the Bcl-2 family into Bid which initiates the mitochondrial apoptosis pathway followed by the release of cytochrome c and Smac/DIABLO from the mitochondria (64). 

It has been demonstrated that crocin pretreatment inhibited MCF-7 cell proliferation through induction of apoptosis accompanied with extensive DNA damage. In this regard, crocin may induce apoptosis in MCF-7 cells via Bcl-2 down-regulation and caspase-3 dependent pathways. Caspase-3 is a well-known downstream adaptor caspase that can be proteolytically activated by caspase-9 or caspase-8 via mitochondrial or cell death receptor signaling pathways, respectively (65, 66). It is well-known that the side effects associated with chemotherapy methods against breast cancer are inevitable. Some of these side effects are systemic toxicity, immunosuppression system, and cardiac toxicity (67). Combination chemotherapy is one of the most important methods which can increase the effectiveness of this route. In the other words, combination of very low toxic phytochemical agents with common chemotherapeutic drugs, reduces side effects while increasing the effectiveness of these drugs through the synergic effect (68).

It has been shown that growth of breast cancer cells treated with combination of crocin and hyperthermia is markedly reduced in a dose and time dependent manner. However, crocin had no significant cytotoxic effect on normal cells. This treatment decreases colony formation of cancer cells by up to 94%. Furthermore, combination of crocin with hyperthermia has a more apoptotic effect than crocin alone (50). Synergistic apoptotic effect of crocin and paclitaxel and also crocin and gamma radiation have been studied on breast cancer MCF-7 cell line (51).

Cyclin D1 plays an important role in breast cancer development and progression through cyclin-dependent kinase (CDK)-dependent and CDK-independent interactions (69). The overexpression of cyclin D1 and activation of CDKs in G1 phase may be the key factors for shortening the G1 phase, increasing the cell proliferation rate and oncogenesis (70). Cyclin D1 also contributes to inactivation of tumor suppressor protein, retinoblastoma protein, in a CDK-dependent manner that can enhance tumor progression (69). It also plays an important role in mitogenic effect of estrogen on breast cancer cells independently of CDK (71). It has been reported that cyclin D1 overexpression causes breast cancer (72) and has an inverse relationship with survival (73).

Effect of crocin on N-Nitroso-N-Methyl urea-induced breast cancer may be related to its potential suppression of cyclin D1 and p21Cip1 overexpression both in mRNA and protein levels, and therefore suppression of tumor growth, and induction of cell cycle arrest in this type of breast cancer cells through a p53-dependent manner (74). p21, a CDK inhibitor, has been known as a downstream target of tumor suppressor p53 (75). However, the coexpression of cyclin D1 and p21 protein is required for the initial steps of tumor development. The overexpression of cyclin D1 and p21 is reported in human cancers and correlated with a high tumor grade and poor prognosis (76). 

It is possible that the inhibitory effect of crocin on cancer cell division is related to microtubule stabilizing (77). Microtubules are involved in different functions in eukaryotic cells including mitosis, axon extension (78), cell migration (79), and signal transduction (77). Microtubules are highly dynamic cytoskeletal proteins in dividing cells, and play a substantial role in spindle formation during mitosis. There are several microtubule binding agents that have antimitotic and therefore anticancer effects by binding to microtubules and suppressing their dynamicity. These agents are classified into two groups of microtubule-destabilizing and microtubule-stabilizing agents that can inhibit and enhance microtubule polymerization, respectively. Both of these agents can suppress microtubules dynamics. On the other hand, these agents kinetically can inhibit any changes in microtubule polymer mass and therefore can block the mitosis (79).

Crocin can enhance the polymerization of microtubules by increasing the light scattering signal of tubulin. This effect is comparable with paclitaxel (taxol), a microtubule-stabilizing agent, that has been used for chemotherapy in various types of cancer (80). It also is a microtubule-destabilizing agent by depolymerization of the interphase and spindle microtubules. In addition, crocin binds to purified tubulin and inhibits the assembly of reconstituted microtubules. Therefore, crocin can inhibit microtubule polymerization either by inducing the formation of tubulin oligomers or by producing defective microtubules (81).

***Lung cancer***

Lung cancer is one of the most common human cancers and has the highest rate of mortality all around the world (82). Lung tumors can be classified into two histological categories: non-small cell lung cancer (NSCLC) and small cell lung cancer (SCLC). NSCLC is the most common type and accounting for about 80% of all lung cancers and associated with poor prognosis. NSCLC includes three main subtypes (adenocarcinoma, squamous cell, and large cell carcinoma) (83), whereas SCLC is less common and comprising 20% of human lung cancers. The predisposing and risk factors of lung cancer include family history, smoking, age, ionizing radiation exposure, viral infection, and chronic inflammatory diseases such as pulmonary fibrosis and chronic obstructive pulmonary disease (COPD) (84, 85). 

It has been demonstrated that saffron decreases proliferation of lung cancer A549 cells, in a dose- and time-dependent manner and induces apoptosis and activates caspase pathways in these human alveolar basal epithelial cells (86). Furthermore, saffron has been shown to affect the rate of proliferation and apoptosis of lung adenocarcinoma cell lines, NSCLC A549 and SPC-A1 cells treated with crocin. In this regard, crocin inhibits cell proliferation rate in human lung adenocarcinoma cells and increases sensitivity of these cells to cisplatin and pemetrexed through induction of G0/G1 arrest, and also through apoptosis by p53 and Bax up-regulation, but Bcl-2 down-regulation (87). 

***Prostate cancer***

Prostate cancer is one of the most important cancers and the major cause of death among men worldwide. Due to slow tumor growth and metastasis, about 90% of all deaths caused by this cancer are observed at the time of diagnosis (88).

Antiproliferative effects of saffron extract and crocin have been studied on prostate cancer cell lines. Crocin inhibits progression of the cell cycle by down-regulating the expression of cyclin D1 in a dose dependent manner. Furthermore, crocin induces apoptosis in human prostate cancer cell lines, partly via an intrinsic pathway of apoptosis by activation of caspase-9 (89).

Reduced intercellular adhesiveness is one of the most important features of cancer cells for initiating their invasive and metastatic behavior (90). Epithelial–mesenchymal transition (EMT) is a biological process that allows epithelial cells to acquire mesenchymal, fibroblast-like properties, reduced intercellular adhesion, and increased motility (91). It has been indicated that invasive and metastatic behavior of epithelial cancer cells may be critically dependent on the acquisition of EMT features by these cells (92). EMT is associated with reduced E-cadherin, increased N-cadherin, β catenin expression, contribution to increased tumor cell motility, and invasive properties. This molecular profile has recently been reported in several tumors (92, 93). Another important aspect of EMT is overexpression of proteolytic enzymes of the extracellular matrix (ECM) such as matrix metalloproteinase and urokinase-type plasminogen activator (53, 94). 

In this regard, the effects of crocin, crocetin, and saffron extract were evaluated on tumor growth of two aggressive prostate cancer cells (PC3 and 22rv1). These carotenoids can reverse EMT and also significantly reduced N-cadherin, beta-catenin expression, increased expression of E-cadherin, and modulate matrix metalloproteinase 2, 9 (MMP2 and MMP9), and urokinase-type plasminogen activator expression/activity in tumor cells (53).

***Liver cancer***

Hepatocellular carcinoma (HCC) is the main form of liver cancer. Hepatitis B and C viruses and contamination of foodstuff with aflatoxins, a type of mold that is considered a human carcinogen, are the main causes of HCC in almost all low-income countries. Alcoholic cirrhosis, tobacco smoking, and diabetes are also related to an excess risk of HCC (95).

The administration of crocin leads to inhibition of cell proliferation and also induction of apoptosis in the cancer cells. Crocin also inhibits Nf-kB in hepatocytes, suppresses S and G2/M phases of the cell cycle, induces apoptosis, and down-regulates inflammation in HepG2 cells (46).

There is a telomerase activity in 85 to 90 percent of human tumors that contribute to continued growth and immortality of tumor cells (96). It has been shown that crocin inhibits telomerase activity of hepatocarcinoma HepG2 cells probably by lowering the expression level of catalytic subunit of telomerase (hTERT) gene. This result implies that crocin increases the immortality rate of hepatic cancer cells by inhibiting telomerase activity (97).

***Cervical cancer***

Cervical cancer is one of the most common neoplastic diseases worldwide (98). 

It accounts for 12% of all female cancers and more than half of cervical cancers occur in developing countries (99). In spite of advancements in treatment, tumor cell resistance to drugs causes recurrence of cancer (100).

Crocin has anticancer effects on human cervical cancer and cytotoxic action on HeLa cell line through shrinking and piknosis in nuclei (56). Liposomes have been administered for a wide range of drug and vaccine delivery applications (101). Crocin liposomal forms have been demonstrated to show enhanced cytotoxic effects compared with crocin in HeLa cells. 

Crocin and crocin liposomal forms induce sub-G1 peak, a reliable biochemical marker of apoptosis, in the flowcytometry histogram of treated cells. These findings indicated that apoptosis through decreasing cell viability is involved in toxicity induction in HeLa cells (102).

Both crocin and crocetin have been demonstrated to activate Nrf2 and up-regulate anti-oxidant response elements (AREs) such as NQO1, NQO2, and HO-1 in HeLa cells (103). However, Nrf2 is a major transcription factor that induces AREs transcription (104). Nrf2 activation also is considered as a protective response against oxidative stress (105). Cancer cells preferably rely on aerobic glycolysis even in the presence of sufficient oxygen to meet the cellular metabolic needs phenomenon termed “the Warburg effect” (106). It seems that this feature protects cells from mitochondrial reactive oxygen species (ROS) (103). Knockdown of lactate dehydrogenase A (LDHA), a key mediator of aerobic glycolysis, increases mitochondrial ROS production associated with decreased cell proliferation (107).

**Figure 3:**
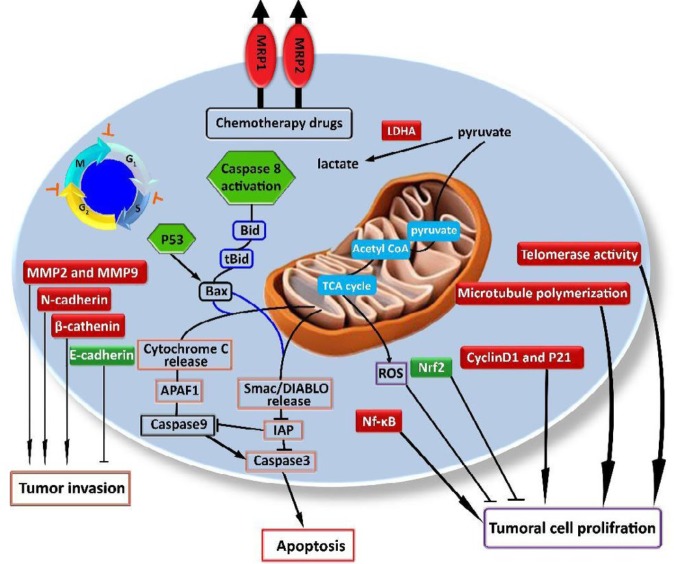
Some important molecular mechanisms involved in the cytotoxic effect of crocin

Knockdown LDHA in neu-initiated mammary tumor cells decreases mitochondrial membrane potentials and cellular ATP levels, and therefore induces oxidative stress and apoptosis in these cancer cells (108). Crocin, slightly, and crocetin, to a greater extent can repress the expression and activity of this key mediator (LDHA) in Hela cells and thus probably induce cytotoxicity in these cells through elevation of ROS production (103). (see Figure 3). 

***Tongue squamous cell carcinoma***

Crocin has a proapoptotic effect and significant decreasing impact on cell viability, growth, DNA, RNA contents, and also on the ratio of RNA/DNA in human tongue squamous cell carcinoma cell line Tca8113. Decreased DNA content can be indicative of DNA damage or suppression of DNA synthesis and eventually inhibition of cancer cell division rate (57).

***Hematologic neoplasms***

Leukemia comprises a heterogeneous group of diseases characterized by the malignant clonal proliferation of blood progenitor cells. These cells primarily develop and expand in the bone marrow and can circulate from there to peripheral hematopoietic tissues. Leukemia leads to a range of systemic symptoms, including anemia, bleeding, and risk of life-threatening infections. It is classified into acute or chronic phases, depending on the clinical course; and or myeloid or lymphocytic, depending on the malignant stem cell of origin (109). The survival rates have improved remarkably over the past decades, largely due to conventional chemotherapy. However, the side effects of these drugs remain significant, further improvements in outcomes will depend on anticancer drugs with high efficacy and low toxicity (110).

According to documents, the anti-leukemic effects of crocin have been investigated in a human leukemia cell line (HL-60 cells) *in vitro* and *in vivo*. Evidence showed that crocin has anti-proliferative and pro-apoptotic effects on these cells and induces cell cycle arrest at the G0/G1 phase, in a concentration and time-dependent manner. Treatment of HL-60 cell xenografted mice with crocin inhibited the tumor weight, size, and Bcl-2 expression and increased Bax expression in these cells (111).

Crocin also promotes Jurkat (human T-cell leukemia cell line) cell apoptosis and inhibits cell growth, in a dose and time-dependent manner. This effect may be related to inhibition of Bcl-2 and promotion of Bax gene expression, which suggests crocin can be used as a suitable clinical agent for the treatment of T-lineage acute lymphoblastic leukemia (T-ALL) (112).

Multiple myeloma (MM), another type of hematologic neoplasm, is characterized by anemia, lytic bone lesions, and elevated M protein in blood or urine and is associated with renal dysfunction (113). The possible cytotoxic activity of crocin on B lymphocytes in human myeloma (U266 cell line) was reported. Crocin declined cell viability of U266 cells to some extent. Crocin also slightly increase the population of DNA fragmented cells and apoptosis in the U266 cell line (114).

Heat shock proteins (HSPs) have been found to be overexpressed in a wide variety of human carcinomas, including both solid tumors and hematological malignancies (115, 116). Three main types of HSPs such as HSP90, HSP70, and small HSPs (117) are expressed at a low level under normal physiological conditions, although environmental stresses could increase their expression (118). Crocin had no significant effect on the expression of HSPs70 and HSP90 in the U266 cell line (114).

***Ovarian cancer***

Ovarian cancer is the seventh most common cancer in women accounting for almost one-third of invasive malignancies of the female genital organs and has remained the leading cause of death from gynecological cancers (119). Multidrug resistance (MDR) is one of the most important mechanisms through which various cancers resist chemotherapy drugs. Overexpression of the membrane efflux proteins has a major role in occurrence of this mechanism (120). Several members of the multidrug resistance**-**associated proteins (MRPs) family especially MRP1 and MRP2 are able to transport anti-cancer drugs out of the cells and present in many different types of tumors and therefore assumed to cause MDR (121).

The cytotoxic activity of crocin in ovarian cancer was reported in human ovarian carcinoma cell lines A2780 and its cisplatin-resistant derivative A2780/ RCIS cells (MRP2-overexpressing cell line). This effect can be attributed to reduction of cell proliferation by crocin in a dose-dependent manner and accompanied by marked reduction of MRP1 and MRP2 gene expression at the mRNA level in A2780/RCIS cells (122). Crocin significantly inhibits the growth of HO-8910 cells in the G0/G1 phase. It can promote apoptosis in these cells, most likely by increasing p53 and Fas/APO-1 expression (123)*.*

***Pancreatic cancer***

 Pancreatic cancer is one of the most lethal malignancies in the world (124, 125). The incidence of pancreatic cancer has been increased over the last decades (126). The cytotoxic effect of crocin in a pancreatic cancer cell line (BxPC-3) was reported and revealed that crocin can induce apoptosis and G1-phase cell cycle arrest of BxPC-3 cells, while decreasing cell viability in a dose and time dependent manner through induction of apoptosis and DNA fragmentation (127). 

***Gastric cancer***

Gastric cancer is a malignant epithelial tumor that arises from neoplasia in the glandular epithelium of the gastric mucosa (128). However, the incidence of gastric cancer has declined worldwide, it used to be the second leading cause of cancer related deaths and the fourth most diagnosed cancer throughout the world (129). Surgery, radiation, and chemotherapy are treatments that are commonly used for gastric cancer (128). It is mentioned that crocin treatment significantly decreased cell viability in a dose, and time dependent manner in the gastric adenocarcinoma (AGS) but not in HFSF-PI3 cells. Moreover, treatment with crocin increased caspase activities, sub G1 apoptotic fraction, and Bax/Bcl-2 ratio, which indicates apoptosis contributed to crocin- induced AGS growth inhibition (130). 

**Table 1:** The effects of crocin on various cell lines (cytotoxic) and animal model (anti-tumor)

References	Cell type	Concentration or dose/duration/ animal	Study design	Target system
_(_ 33_)_	HCT-116, HT-29 & SW-480	0.03,0.1,0.3 & 1.0 Mm	Cell line study	Colon
_(_ 34_)_	HCT116 & HCT116 p53−/−	10 Mml 24 & 48 hr/rat	Cell line study	Colon
_(_ 48_)_	-	50,100 & 200 ppm/15 weeks in diet/mice	Colonic adenocarcinoma induction by DSS	Colon
_(_ 49_)_	MCF-7	10,25,50 µg/ml/24 hr/human	Cell line study	Breast
_(_ 50_)_	MDA-MB-468	0-5 mg/ml/0-72 hr/human	Cell line study	Breast
_(_ 51_)_	MCF-7	2.5 mg/ml/48 hr/human	Cell line study	Breast
_(_ 52_)_	A549 & SPC-A1	1,2,4,8,16 mg/ml/human	Cell line study	Lung
_(_ 53_)_	PC3 & 22rv1	200 mg/kg /PO/ 5 day/week/human	Cancer cells	Prostate
_(_ 54_)_	LnCaP, 22rv1, CRW PC3 & 145 DU	0.1-4 Mm/48 hr/human	Cell line study	Prostate
_(_ 55_)_	HepG2	3 mg/ml/48 hr/human	Cell line study	Liver
(56)	Hella	Crocin: 1, 2 & 4 mM crocin liposomal forms: 0.5 & 1 mM/24, 48 hr & 72 hr/ human	Cell line study	Cervix
(57)	Tca8113	0.01, 0.2 , 0.4 & 0.8 mM 24, 48, 72, & 96 hr/human	Cell line study	Tongue

Crocin inhibits telomerase activity, microtubule polymerization, cyclin D1 and p21 over expression, NF-kB expression and activation, and MRP1 and MRP2 expression contribute to tumor cell proliferation. Crocin also inhibits lactate dehydrogenase A (LDHA) and therefore causes increased production of mitochondrial ROS. Crocin can induce apoptosis through activation of caspase 8 and increasing p53 expression. It down-regulates matrix metalloproteinase 2 and 9 (MMP2 and MMP9), N-cadherin and beta-catenin expression, which are involved in tumor invasion and metastasis. Tumor invasion is inhibited by crocin through increasing E-cadherin expression. Cell cycle suppression at G1 and G0/G1 phases, S and G2/M phases are also induced by crocin in cancer cells. (legend of symbols: arrows indicate activation. truncated lines indicate inhibition. Red shapes indicate molecules or reactions down-regulated or inhibited by crocin. Green shapes indicate molecules or reactions up-regulated or activated by crocin. Short truncated orange lines around the cell cycle diagram indicate inhibition by crocin in certain phases)

## Conclusion

Natural products are of particular interest as preventive agents due to their low toxicity and potent efﬁcacy. Crocin belongs to carotenoids, which are synthesized in subcellular organelles of plants and are used to prevent and treat various diseases. The antitumor mechanisms of crocin are apoptosis, inhibition of cell proliferation, and cell cycle progression, reduction of MRP1 and MRP2 gene expression, inhibition of telomerase activity, microtubule polymerization, and suppression of cyclin D1 and p21Cip1 overexpression. Moreover, this carotenoid can reduce N-cadherin, beta-catenin expression, increase expression of E-cadherin, and down-regulate matrix metalloproteinases 2 and 9, and urokinase-type plasminogen activator expression/activity in tumor cells. Furthermore, it decreases the mRNA expression of certain proinﬂammatory cytokines associated with cancer. Although several hypotheses have been put forward, the precise mechanisms underlying crocin effects against cancer are not clear as yet.

## Conflicts of Interest

The authors declare that there are no conflicts of interest.
